# Design and Evaluation of Passive Shoulder Joint Tracking Module for Upper-Limb Rehabilitation Robots

**DOI:** 10.3389/fnbot.2018.00038

**Published:** 2018-07-27

**Authors:** Kyoung-Soub Lee, Jeong-Ho Park, Jaewon Beom, Hyung-Soon Park

**Affiliations:** ^1^Department of Mechanical Engineering, Korea Advanced Institute of Science and Technology, Daejeon, South Korea; ^2^Department of Physical Medicine & Rehabilitation, Chung-Ang University Hospital, Seoul, South Korea

**Keywords:** gravity compensation, joint tracking, motion capture, passive mechanism, rehabilitation robotics

## Abstract

As the number of people suffering from shoulder movement disabilities increases, there is a rising demand for shoulder rehabilitation. The natural motion of the shoulder joint [glenohumeral (GH) joint] includes not only three-degrees-of-freedom (DOF) rotation but also three-DOF translation of the joint center due to simultaneous motion of the shoulder girdle. If the motion of the shoulder girdle is restricted, then the arm cannot be raised above a certain posture. This paper presents a passive shoulder joint tracking device that allows three-DOF translation of the shoulder joint while compensating for gravity. The single-DOF vertical tracker with a constant-force spring compensates for the gross weight of the user's arm, the upper limb rehabilitation device, and the tracker itself while allowing vertical tracking motion. The two-DOF horizontal tracker consists of two linear guides arranged perpendicular to each other. The tracker freely follows the shoulder joint in the horizontal plane. The effect of using the passive shoulder joint tracking device was evaluated by means of experiments by combining two popular commercial upper limb rehabilitation apparatuses with the proposed tracker. Nineteen subjects (8 healthy persons and 11 patients with shoulder impairments) participated in the evaluation study. The movement of the GH joint and the interactive force between the subject and the commercial rehabilitation device were analyzed when subjects made the following shoulder movements: flexion/extension and abduction/adduction. The improved tracker allowed a greater range of motion and reduced interaction. The tracker can be combined with existing commercial rehabilitation devices for more natural shoulder movement during rehabilitation tasks.

## Introduction

In recent years, the number of people suffering from various shoulder movement disabilities due to neurological diseases and orthopedics has been increasing. Stroke, one of the most prevalent neurological diseases that affect the elderly, frequently causes shoulder subluxation, that is, misalignment of the joint (Smith et al., 1982; Zorowitz et al., 1996; Ikai et al., 1998; Turner-Stokes and Jackson, 2002). Orthopedic diseases including shoulder impingement syndrome and rotator cuff injuries are also on the rise. Repeated overhead shoulder motion during intense exercises such as swimming, tennis, and lifting can cause shoulder impingement syndrome. Patients suffering from this syndrome feel pain whenever they try to raise their arms above a certain level (Fu et al., 1991). It can lead to chronic illness without systematic management and treatment (Ingber, 2000). When a rotator cuff injury occurs, it is difficult for patients to suddenly raise their arm above their head and maintain their posture. In such cases, therapists must restore the function of the patients' shoulder complexes by recovering muscle strength and range of motion (ROM) of the joint (Brukner, 2012). Patients' shoulders tend to deteriorate over time because they do not use the affected side (Chakravarty and Webley, 1990; Chard et al., 1991). To overcome this problem, they need regular rehabilitation therapy to reduce pain and restore function (Koh and Lim, 2013).

The kinematic movement of the shoulder is the most complicated one in the human body as it involves the movement of three bones and four joints: the clavicle (collarbone), scapula (shoulder blade), humerus (upper arm), sternoclavicular (SC) joint, acromioclavicular (AC) joint, glenohumeral (GH) joint, and scapulothoracic joint. The bones are connected with multiple muscles and move simultaneously; only the clavicle is attached to the thorax. The SC joint attaches the clavicle and manubrium of the sternum. The other side of the collarbone is attached to the acromion (the highest part of the scapula) at the AC joint. The scapulothoracic joint is a sliding joint between the medial border of the scapula and the torso. The shoulder joint is usually defined as the GH joint, which is the ball-and-socket joint between the humerus and scapula. The shoulder joint has three-DOF rotational motions—flexion–extension (motion in the sagittal plane), abduction–adduction (motion in the coronal plane), and medial–lateral rotation (motion in the transversal plane). The horizontal abduction–adduction is the upper arm rotation in the transversal plane. As the scapula is connected to the trunk through a clavicle and a sliding joint, three-DOF movements about the trunk occur: protraction–retraction (movement toward the spine), elevation–depression (movement upward and downward), and upward–downward rotation. These movements induce the translational movement of the GH joint. To allow wide ROM of the shoulder joint, the translation of the GH joint center (CGH) is essential (Dvir and Berme, 1978; Maurel and Thalmann, 1999; McClure et al., 2001; Borstad and Ludewig, 2002; Forte et al., 2009). The movement of the shoulder girdle (clavicle and scapula) is more important when raising the arm above the shoulder than the arm motion below the shoulder. As the arm is raised, the ratio of scapulothoracic rotation (rotation of the scapula relative to the torso) to GH rotation (rotation of humerus relative to the scapula) generally increases, but the ratio varies depending on the subject (Culham and Peat, 1993). If the movement of the shoulder girdle is restricted, then the arm cannot be raised above a certain posture. Therefore, translation of the GH joint is essential to enable the arm to be raised high. If the translational motion is limited by external forces, then the ROM of the shoulder joint is limited and it may cause pain in the joint (Esmaeili et al., 2011).

For instrumented shoulder rehabilitation, various robotic devices, ranging from passive mechanisms such as Wrex (Haumont et al., 2011) or single-DOF continuous passive motion (CPM) machines to multi-DOF robots such as Armeo (Hocoma AG, Volketswil, Switzerland) (Colomer et al., 2013; Calabrò et al., 2016), have been commercialized. However, shoulder translation was not considered when most of these devices were created. They limit the ROM of the GH joint entirely (or partially) and interfere with the natural shoulder motion. On the other hand, it has been reported that several advanced robots allow CGH translation (Ball et al., 2007; Perry et al., 2007; Nef and Riener, 2008; Park et al., 2008). These robots use one or more additional actuators to support the three-dimensional translational motion of the CGH. The additional actuators inevitably require high torque capacity to drive high inertia. As high-capacity actuators are excessively bulky, heavy, and expensive, their use has meant that robots are rarely used in clinics.

The rehabilitation of natural shoulder motion in clinics would require a compact device to allow the CGH translation. In our laboratory, a passive shoulder joint tracker, featuring a lightweight and simple mechanism, using a compressive spring was developed (Park et al., 2016). The compressive springs lift the tracker to follow the vertical motion of the CGH, which compensates for the combined weight of the user's arm and the device. In addition, a two-link mechanism follows the horizontal translation of the CGH. This device was evaluated by CGH estimation by using an experiment to capture two different shoulder motions (flexion and abduction) with the aid of five healthy male subjects. In comparison to the original J-Wrex without the tracker, J-Wrex combined with the tracker improved the translational ROM of CGH.

This paper presents a modified design of the tracker and its experimental performance for evaluating the CGH motion and the applied force using two devices: J-Wrex (Jaeco orthopedics, Hot Springs, USA) and CPM (Centura Shoulder CPM, Kinetec, France). Eight healthy subjects and 11 patients with upper limb impairment participated in the evaluation. The tracker increased the ROM of the upper limb and reduced the interactive force between the device and the patient.

The rest of this paper is organized as follows. The section on “Design of Device” describes the design of the passive shoulder joint tracker. The section on “Design of Experimental Evaluation” describes the experimental setups, data recording protocol and data analysis method. The “Results” section presents the experimental results, and the section on “Discussion and Conclusion” concludes the paper and discusses the future work to improve the device and the dataset.

## Design of device

The first prototype of the tracker was developed as part of our previous work (Park et al., 2016). This prototype consisted of a horizontal tracker with a two-link mechanism and a vertical tracker supported by a compressive spring, consisting of small spring segments connected in series. The motion of scapular in protraction/retraction causes the GH joint to move in a transversal plane. The tracker in medial–lateral direction mainly allows the movement of GH joint and the anterior–posterior direction is also involved in the movement. The elevation/depression of the scapula that causes the GH joint to move upward/downward can be primarily tracked by the vertical tracker. The movements of the scapula and the CGH depend on the particular situation: the amount of external resistance, stretching condition, and disease (McQuade and Smidt, 1998; Wang et al., 1999; Lin et al., 2006). Therefore, a passive shoulder joint tracker that could adapt to each situation was designed to allow the upper limb to undergo passive motion. Prior to the design of the horizontal tracker, the horizontal workspace of the GH joint was roughly identified through a pilot study based on measurement of the trajectory of the acromion process (the bony landmark closest to the GH joint). This study involved five healthy male subjects who performed five shoulder motions three times: frontal raise, raise at side, swing at side after frontal raise, scaption (of 45°), and touching head. The position of the CGH was estimated by subtracting a constant vector from the location of the acromion process which is the bony landmark closest to the CGH. The vector was obtained by the anatomical structure of the shoulder (Murray, 1999). The study revealed that the shoulder joint moved posteriorly and medially, whereas the subjects raised their arms and moved in reverse as the subjects lowered their arms (Park et al., 2015). Based on these findings, an elliptical area was identified by overlapping the horizontal trajectories of the CGH with the exclusion of a few outlying trials. The dimensions and the initial posture of the tracker were determined to ensure that it covers the workspace.

In Park et al. (2016), two design modifications—the structure of the horizontal tracker and change of the spring—were suggested after evaluation of the tracker. On the basis of the modification, an improved prototype of the tracker was developed. First, the two-link mechanism of the horizontal tracker was replaced by two linear guides that are aligned perpendicularly to each other. As the CGH moves almost linearly and the linear guides have a high-rated load, the linear guides offer an advantage over the two-link mechanism in following the trajectories of the CGH in the horizontal plane. Second, the serial connection of the compressive springs was replaced by a constant-force spring to achieve a constant compensation force. In the first prototype, the compensation force varied with the tension length because of the nature of the coil spring. Because of the difference between the gravity and the compensating force, a downward force is generated as the device moves upward. This restricts the ROM of the shoulder and the tracker. In addition, as the compensation force of the modified tracker does not vary with height, it is simpler to adjust the initial height for each patient. Details of these modifications are explained in the following sections.

### Two-DOF horizontal tracker

**Figure 1:**
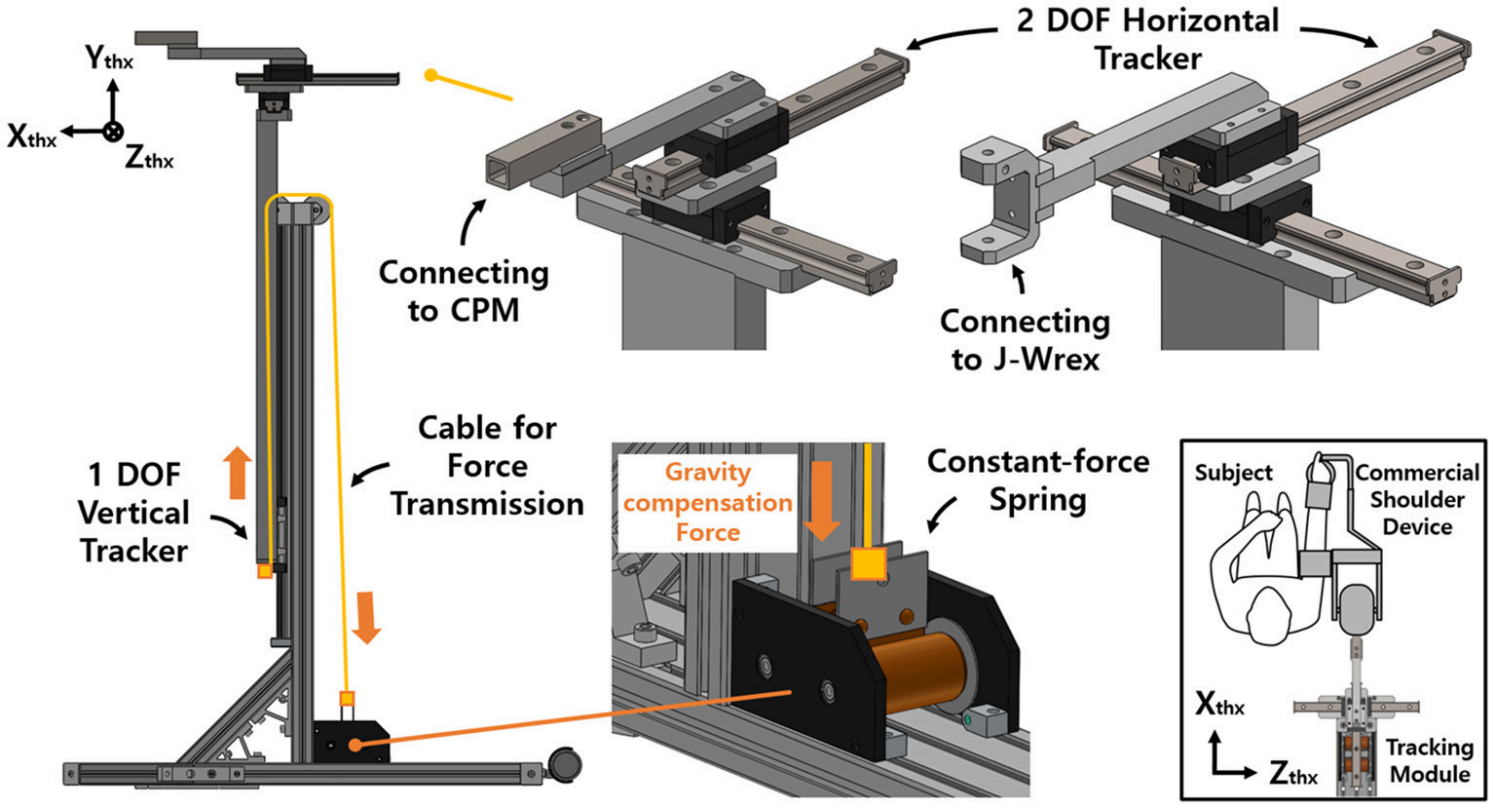
Design of the passive shoulder joint tracking module and the coordinate system.

Two linear guides were aligned perpendicularly to each other to enable horizontal tracking of the GH joint. The anterior–posterior linear guide was mounted on the medial–lateral linear guide. The linear guides have a ROM of 153 and 213 mm in the medial–lateral and anterior–posterior directions, respectively. The tracker enables any translation of the CGH with even manipulability in the 213 and 153 mm space. The linear motion guide has the advantage of being able to move linearly through the ball in the block with almost no friction. It minimizes the force on the shoulder due to frictional force when moving horizontally. Any commercial rehabilitation device for the upper limb can be attached to a sliding block of the anterior–posterior linear guide by way of a custom-designed part. In this regard, J-Wrex and CPM were used as the commercial device in our experimental evaluation. Prior to the attachment, the upper limb apparatuses of the devices were isolated from their frame parts and reconnected by using the custom-designed parts, as shown in Figure 1.

### Single-DOF vertical tracker with gravity compensation

The vertical tracker features a simple gravity compensation mechanism using a constant-force spring. This tracker is a linear guide unit designed to support the large moment generated by the weight of the device and the user's arm. The spring pulls up the tracker to provide an upward force, compensating for the gross weight of the user's arm, the tracker, and the rehabilitation device.

The previous gravity compensation mechanism using the coil spring was able to compensate for the force required by adjusting the spring constant according to the user's upper limb (Park et al., 2016). However, the characteristics of the spring resulted in an increase in the compensation force as the tension length increased. In addition, because of the difference between gravity and the compensation force, a downward pressing force was generated as the shoulder moved upward.

**Figure 2:**
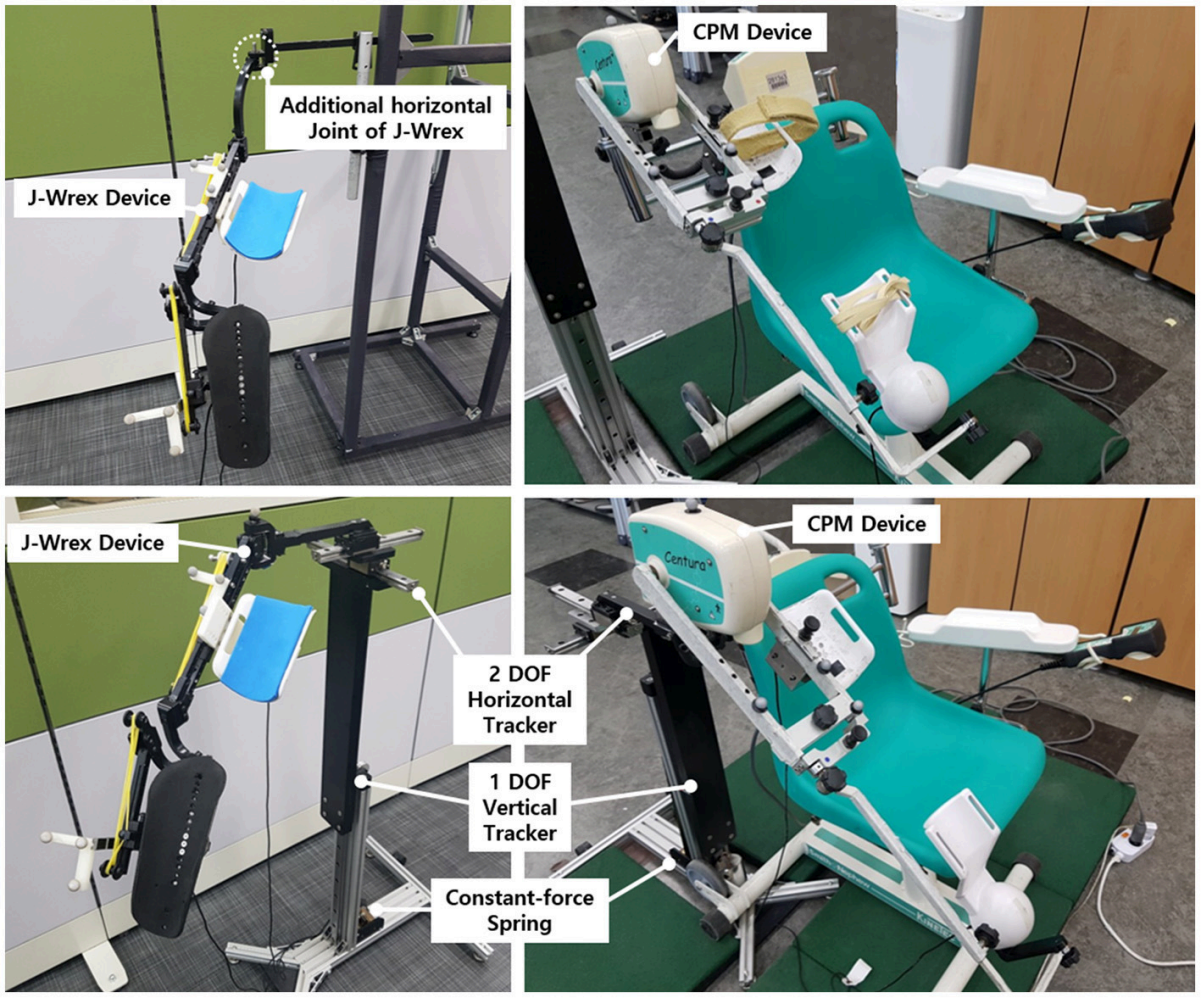
J-Wrex **(Left)** and CPM **(Right)** with **(Lower)** and without **(Upper)** the tracker.

Accordingly, an improved mechanism was designed using a constant-force spring with a constant-restoring force regardless of the tensile length. A constant-force spring is a type of leaf spring that is bent with a certain curvature, and the load generated when it is stretched in a straight line is constant even after reaching the maximum load (after the drum is rotated 1/2 turn). In addition, as the structure is designed for use in a small-diameter drum, the space for routing the spring is not needed (Figure 2).

The force of the spring was determined by considering the total weight of the rehabilitation device, the tracking device, and the arm. This enabled us to determine the compensating force for J-Wrex and CPM, respectively.

The mass of the J-Wrex device was 1.5 kg, and the total mass of the three-DOF tracker was 2.38 kg. The mass of one arm corresponds to 4–5% of the total mass of the human body (Ma et al., 2011). Our design compensated for the gravity force of ~2 kgf in consideration of the weight of the arm by using a spring with a load of 5.7 kgf. The mass of the actuating part of CPM was 4.2 kg, and the total mass of the three-DOF tracker was 3.4 kg. Similarly, considering the weight of the arm, two constant-force springs that compensated for a force of 4.7 kgf were used to hold a load of 9.4 kgf. The compensation force was adjusted by adding a counter mass according to each patient's mass. The mass of each part is provided in Table 1.

**Table 1:** Setting of the springs.

**Combined device**	**Spring Force (kgf)**	**Mass of tracker and device (kg)**
J-Wrex	5.7	2.38 + 1.5 (tracker + J-Wrex)
CPM	9.4	3.4 + 4.2 (tracker + CPM)

## Design of experimental evaluation

The tracker was evaluated based on a three-DOF motion capture experiment of the two rehabilitation devices, J-Wrex and CPM, and two basic shoulder motions: flexion/extension and abduction/adduction. The experiment was performed under three experimental conditions: free motion without any device (free motion), motion supported by device combined with the tracker (w/ tracker), and motion supported by only the existing device (w/o tracker). In the experiment using the CPM, free motion was not performed. Eight healthy subjects and 11 patients suffering from upper limb impairments participated in this experiment. The characteristics of the patients are listed in Table 2. Each group of subjects gave written consent approved by the International Review Board (IRB) at the Korea Advanced Institute of Science and Technology (KAIST) and Chungnam National University Hospital (CNUH), respectively (KAIST: KH2015-08, CNUH: 2015-10-019-007). The motion capture data was used to estimate the trajectory of the CGH. The force sensors under the wearing part of the upper arm and forearm measured the interactive force between the arm and the device. The CGH motion was compared under three conditions. The interactive force and angle difference between the upper arm and the device were analyzed under the two conditions—w/ the tracker and w/o the tracker—in which the device was used. The comparison of the results obtained for the maximum arm elevation angle, CGH motion, interactive force, and angle difference enabled the evaluation of the ability of the tracker to allow the translation of CGH.

**Table 2:** Characteristics of patients.

**Device**	**Gender**	**Disease**
CPM	M	Guillain-Barre syndrome
CPM/J-Wrex	F	Left olecranon fracture (with shoulder stiffness)
CPM/J-Wrex	F	Left clavicle mid-shaft fracture
CPM^*^	M	Left middle cerebral artery territory infarction
CPM/J-Wrex	M	C4-5-6 fracture
CPM/J-Wrex^*^	M	Right fingers near amputation (with shoulder stiffness)
CPM	F	Right distal radius fracture (with shoulder stiffness)
CPM/J-Wrex	F	Left fingers fracture (with shoulder stiffness)
CPM/J-Wrex	F	Left proximal humerus fracture
J-Wrex	M	Right ulnar & distal radius fracture
J-Wrex	F	Left distal radius fracture (with shoulder stiffness)

* *Patients who were unable to raise their arm over 120°*.

### Motion capture and force sensor set-up

**Figure 3:**
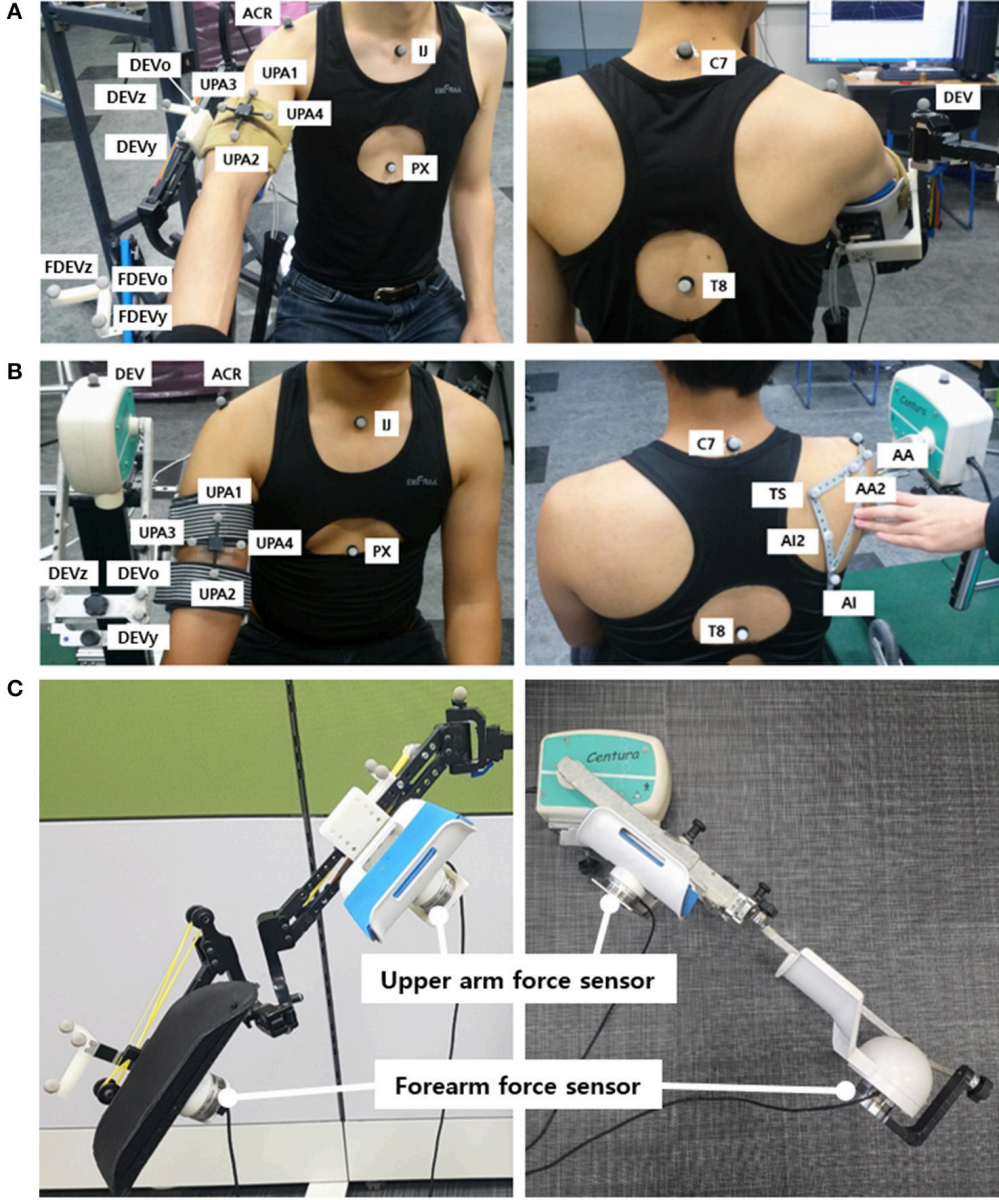
Experimental setup: Marker placement for motion capture experiment of upper limb for **(A)** J-Wrex and **(B)** CPM, **(C)** Location of force sensors (The participants have agreed to publication of this image).

The motion capture experiment was carried out with the use of VICON motion capture systems (Vicon Motion Systems Ltd., Oxford, UK). At KAIST, 8 cameras captured the motion of the healthy subjects, and at CNUH, 12 cameras captured the motion of the patients. The motion and the force data were sampled at the rate of 100 Hz. A total of 18–21 markers were utilized to measure the torso, scapula, upper arm, and the device (Figure 3). Four markers (C7, T8, IJ, and PX) were attached to the seventh cervical vertebra, eighth thoracic vertebra, incisura jugularis, and processus xiphoideus, respectively. If the T8 marker was concealed by the chair or device, then this marker was reconstructed using the T3 marker located on the third thoracic vertebra. Three markers (TS, AA, and AI) represented the trigonum scapulae, angulus acromialis, and angulus inferior, respectively. Placement of these seven markers followed the recommendation of the International Society of Biomechanics (ISB) (Wu et al., 2005). The motion of the scapula was measured with a scapula locator as the markers attached to the skin cannot measure motion. The three markers for the scapula were attached to each corner of the locator. Two additional markers (AA2 and AI2) on the locator were used to prevent situations in which the scapula markers are concealed by the device. One experimenter palpated the scapula of the subject before the experiment and customized the shape of the locator. Four more markers for the upper arm (UPA1-4) were arranged in a cross-shaped manner. UPA 1 and UPA 2 defined the longer axis of the cross, which was aligned with the longitudinal axis of the upper arm, and UPA 3 and UPA 4 defined the shorter axis. To measure the motion of the tracker and the direction of the force sensors, further markers (DEV, UDEVo-x-y, and FDEVo-x-y) were attached along the upper arm and forearm braces. DEV was attached to the end-tip of the horizontal tracker, which is the nearest point of the device to the shoulder. In the experiment using CPM, only three markers were required to set up the coordinates of the device, as the directions of the two braces coincided. The last marker (ACR) was attached to the skin right above the acromion process of the scapula. The position of this marker was compared with the estimated position of the CGH to validate the estimation result.

The interactive forces applied to the subjects were measured by attaching two force sensors (wireless F/T sensor, ATI Industrial Automation, USA) under the upper arm brace and the forearm brace. They were placed between the brace and the device to measure the interactive force between the device and the arm. The values recorded by the two sensors were converted to the upper arm coordinate system.

### Experimental protocol

The experiment was repeated three times for each motion and experimental condition. The flexion/extension and abduction/adduction motions were conducted in sagittal plane and coronal plane, respectively. The experiment consisted of two steps: capturing static postures and capturing motions. The static postures were used to estimate the GH joint using the position of the scapula without skin movement effect. While capturing the static postures, the subjects maintained three postures: resting the lowered arm at the side, raising the arm as high as possible, and maintaining the arm halfway between these two postures. The experimenter aligned the locator with the subjects' scapula based on palpation. The locator was held for 2 s to measure the postures of the scapula, after which the subjects lowered their arms again and rested for a short while until the next trial. Later, they raised their arms as high as possible and lowered their arms again to the resting posture for capturing the motions. For the experiment using the tracker, the device was rotated slightly to avoid occlusion of the C7 and T3 markers. Under the two conditions intended to assess the device, namely w/ tracker and w/o tracker, the force sensor measured the applied force between the braces and the arm. The motion capture and the force sensor were synchronized by the experimenter, who pressed the forearm brace with a marker before capturing the motion. Counter masses were added or removed to eliminate any discomfort experienced by the subjects.

### Analysis of translation of the GH joint center

In this paper, the motions of the torso, scapula, and upper arm (humerus) were analyzed to observe the movement of the GH joint, that is, the rotational center of humerus and scapula. Upper arm brace and forearm brace coordinates were used to analyze the forces. The first step of the analysis entailed building coordinate frames of the torso, scapula, upper arm, and two brace segments. The coordinate systems were aligned to global coordinate [Xg (horizontal, forward), Yg (vertical), and Zg (horizontal, left-right)] in the resting condition. The torso coordinates (Xthx, Ythx, and Zthx) were defined as recommended by Wu et al. (2005) using (1). The vector pointing in the medial–lateral direction was obtained by taking the cross product of the two vectors on the sagittal plane. To obtain the vertical vector using four points, Ythx was defined from the midpoint between PX and T8 to the midpoint between IJ and C7. As the T8 was not visible in this experiment, C7 and T3 were used to estimate the location of C7.

(1) $$\{\begin{array}{c} \begin{array}{c} T8\;=\;C7\;+\;2\left(T3-C7\right)\\ \begin{array}{c} Z_{thx}\;=\;\frac{\left(IJ-PX\right)\;\times \;\left(C7-PX\right)}{\left\|\left(IJ-PX\right)\;\times \;\left(C7-PX\right)\right\|}\\ Y_{thx}\;=\;\frac{\left(IJ\;+\;C7\right)/2\;-\left(PX\;+\;T8\right)/2}{\left\|\left(IJ\;+\;C7\right)/2\;-\left(PX\;+\;T8\right)/2\right\|}\\ X_{thx}\;=\;Y_{thx}\;\times \;Z_{thx} \end{array} \end{array}\\ \text{Origin},\;O_{thx}\;=\;IJ \end{array}$$

**Figure 4:**
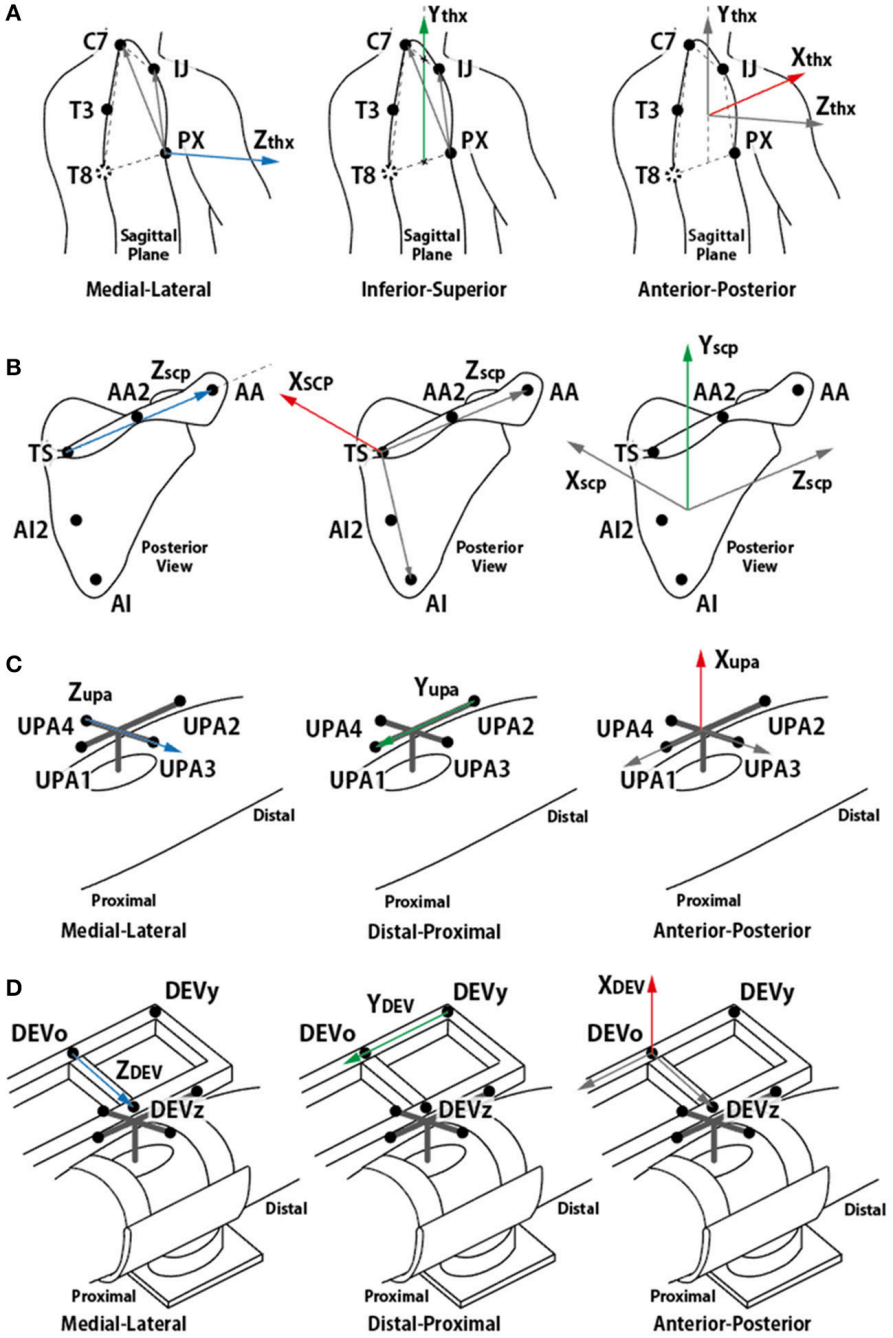
Definition of each segment coordinate frame. **(A)** torso coordinate frame, **(B)** scapula coordinate frame, **(C)** upper arm coordinate frame, and **(D)** device coordinate frame. **(A,B)** followed ISB recommendation (Wu et al., 2005).

To observe the movement of the CGH with respect to the torso, the positions of the markers on the upper arm and scapula (UPA1-4, TS, AA, and AI) were measured and transformed to the positions observed in the torso coordinate frame. The transformed positions were named UPA1-4_thx_, TS_thx_, AA_thx_, and AI_thx_. Subsequently, the scapula coordinate frame (X_scp_, Y_scp_, and Z_scp_), upper arm coordinate frame (X_upa_, Y_upa_, and Z_upa_), and upper arm brace coordinate frame (X_DEV_, Y_DEV_, and Z_DEV_) were defined using (2–4). The definition of the scapula coordinate followed the ISB recommendation as well as the torso coordinate. The meaning of each axis is illustrated in Figure 4.

(2) $$\{\begin{array}{c} \begin{array}{c} Z_{scp}\;=\;\frac{AA_{thx}\;-\;TS_{thx}}{\left\|AA_{thx}\;-\;TS_{thx}\right\|}\\ X_{scp}\;=\;\frac{\left(AA_{thx}\;-\;TS_{thx}\right)\;\times \;\left(AI_{thx}\;-\;TS_{thx}\right)}{\left\|\left(AA_{thx}\;-\;TS_{thx}\right)\;\times \;\left(AI_{thx}\;-\;TS_{thx}\right)\right\|}\\ Y_{scp}\;=\;Z_{scp}\;\times \;X_{scp} \end{array}\\ \text{Origin},\;O_{scp}\;=\;AA \end{array}$$

(3) $$\{\begin{array}{c} \begin{array}{c} Z_{upa}\;=\;\frac{UPA3_{thx}\;-\;UPA4_{thx}}{\left\|UPA3_{thx}\;-\;UPA4_{thx}\right\|}\\ Y_{upa}\;=\;\frac{UPA1_{thx}\;-\;UPA2_{thx}}{\left\|UPA1_{thx}\;-\;UPA2_{thx}\right\|}\\ X_{upa}\;=\;Y_{upa}\;\times \;Z_{upa} \end{array}\\ \text{Origin},\;O_{upa}\;=\;\frac{UPA3\;+\;UPA4}{2} \end{array}$$

(4) $$\{\begin{array}{c} \begin{array}{c} Z_{DEV}\;=\;\frac{DEVz_{thx}\;-\;DEVo_{thx}}{\left\|DEVz_{thx}\;-\;DEVo_{thx}\right\|}\\ Y_{DEV}\;=\;\frac{DEVo_{thx}\;-\;DEVy_{thx}}{\left\|DEVo_{thx}\;-\;DEVy_{thx}\right\|}\\ X_{DEV}\;=\;Y_{DEV}\;\times \;Z_{DEV} \end{array}\\ \text{Origin},\;O_{DEV}\;=\;DEVo \end{array}$$

In some trials, the AI or TS marker was concealed by the chair or a device. Hence, these markers were reconstructed using the AI2 and AA2 markers. This did not change the relative position of the markers on the scapula locator for the same subject. In other trials including the same subject, the relative positions of the concealed markers with respect to the other scapula markers were averaged, and the positions of the corresponding markers were reconstructed.

In addition, the elevation angle of the arm was also calculated. X_upa_, the vector from UPA 1 to UPA 2 (which is aligned with the longitudinal axis of the upper arm) was projected onto the sagittal plane (defined by X_thx_ and Y_thx_) for flexion motion and onto the frontal plane (defined by Z_thx_ and Y_thx_) for abduction motion. The angle was calculated as the angle between the inferior axis of the torso and the projected vector.

**Figure 5:**
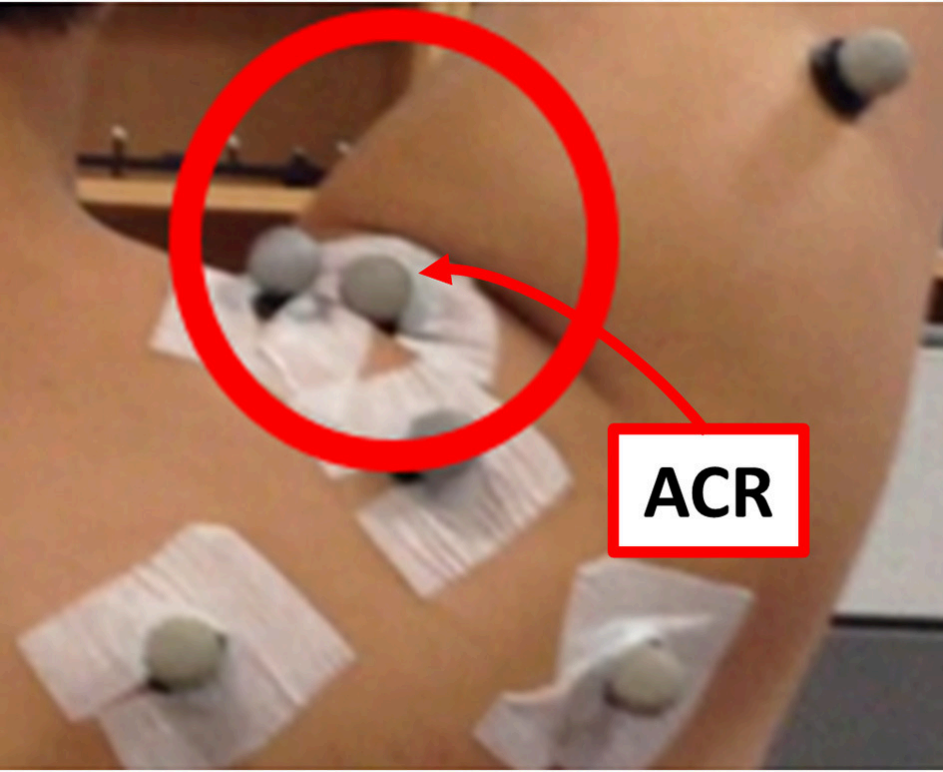
Skin folding observed during the pilot study. The position of the ACR marker was affected significantly by the skin motion.

The last step of the analysis motion is the estimation of the GH joint center. The thickness of the skin and the presence of muscles around the shoulder joint also contribute to the inaccuracy of measurements of the bony landmarks. The pilot study indicated that the position of the ACR marker (right above the acromion process) was affected by the folding skin resulting from raising the arm (Figure 5). Therefore, estimation of the CGH from the motion of segments joined by the shoulder joint is appropriate for measuring its center rather than using bony landmarks for these measurements. The least-squares-based estimation algorithm proposed by Piazza et al. (2004) was used for this purpose. This algorithm was developed based on the fact that there is no relative movement between two segments at the center of rotation. The algorithm, therefore, focuses on finding two points (a fixed point on each of the segments) that minimize the sum of the distance between them at each moment during a certain motion based on the least-squares optimization, as described in (5).

(5) ∂∂qj(∑iei2)=0 (j=1~6)qu=[q1q2q3]: A point fixed to the upper arm referenecedto torso coordinate in (1)       qs=[q4q5q6]: A point fixed to the scapula referenced                               to torso coordinate in (1)Tus: Transformation from the upper arm to scapula segment                                                       coordinate       ei 2=‖[qs1]−usT × [qu1]‖2: Distance between the two points at the i−th data set

The root mean square value of the distance between the optimized points was calculated to check the validity of the estimation. After optimization, two points (past and present mix) were calculated. They are joint centers estimated in each segment coordinate. In this paper, we substituted scapula and the upper arm into the equation to obtain CGH, which is the center of rotation of the segments. It was difficult to measure the position of the scapula during the motion because of the skin movement. Therefore, the position of each segment in the static posture was obtained, and the center position of the upper arm coordinates was used in motion. The position of the joint center was compared with the position of the acromion process marker to check whether the estimated position of the joint center is anatomically plausible. For comparison with the marker attached to the devices, the estimated joint center was transformed back to global coordinates from the torso coordinates.

### Analysis of performance of the tracker

**Figure 6:**
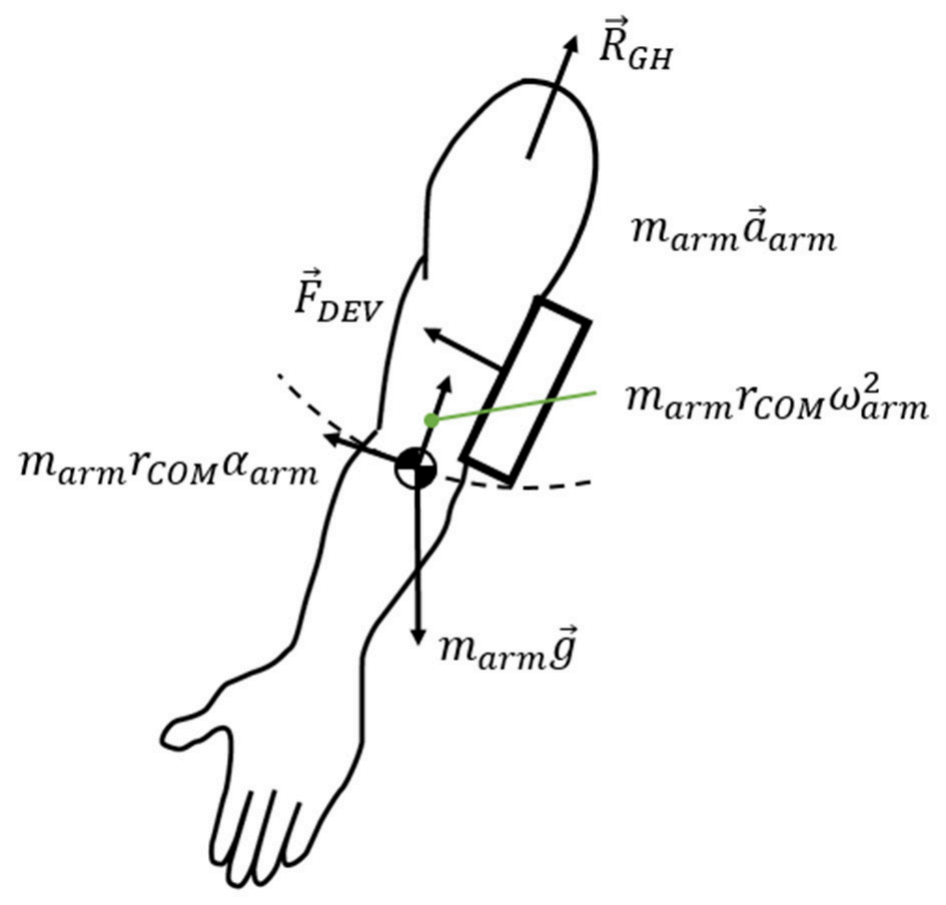
Free body diagram of the arm.

The free body diagram of the arm is shown in Figure 6, and the force equation is derived as follows.

(6) $$R_{GH}\;+\;m_{arm}g\;+\;F_{DEV}\;=\;m_{arm}a$$

where *R*_*GH*_ is the interaction force exerted on the arm at the shoulder joint, *m*_*arm*_*g* is the weight of the arm, and *F*_*DEV*_ is the measured force on the force sensors. *m*_*arm*_*a* is the inertial force of the arm. The force generated under this condition was measured using two sensors: under the upper arm brace and the forearm brace. As J-Wrex has two free joints between the upper arm brace and the forearm brace, the forces measured by the two sensors were transformed into the upper arm brace coordinate system, before being added together. In the CPM experiments, the coordinates of the two sensors matched; therefore, the forces from the two force sensors were summed without transformation.

When using the J-Wrex, the subjects continuously raised their arms at the slowest speed, requiring ~20 s each to raise and lower their arms. In the case of passive exercise using the CPM device, it took the subjects ~2 min to raise their arms and the angular velocity was almost constant. The inertial term, *m*_*arm*_*a*, in (6) is bounded by the magnitude of the vector sum that consists of centripetal force and tangential inertia force. The maximum value from our measurement using *m*_*arm*_ = 3.7 kg and *r*_*COM*_ = 0.32 m is 0.063 N, which is negligible compared to other force terms in (6). Therefore, the force exerted on the shoulder, ignoring the inertial force term, is simplified as follows.

(7) $$R_{GH}\;=\;-m_{arm}g\;-\;F_{DEV}$$

The performance of the tracking module can be analyzed by measuring the difference between the joint angles of the device and the subject's arm. If the device tracks the subject's shoulder joint, then the joint angle difference between the device and the arm is close to zero. When the device does not track the subject's GH joint, the subject's arm slides and tilts inside the brace, causing larger clearance and interaction force between the arm and the brace. In addition, the subject's shoulder joint angle deviates from that of the device. Therefore, the difference in joint angle, ROM of the GH joint, and the interaction force were analyzed to evaluate the performance of the proposed tracking module.

## Results

### Maximum elevation angle

**Figure 7:**
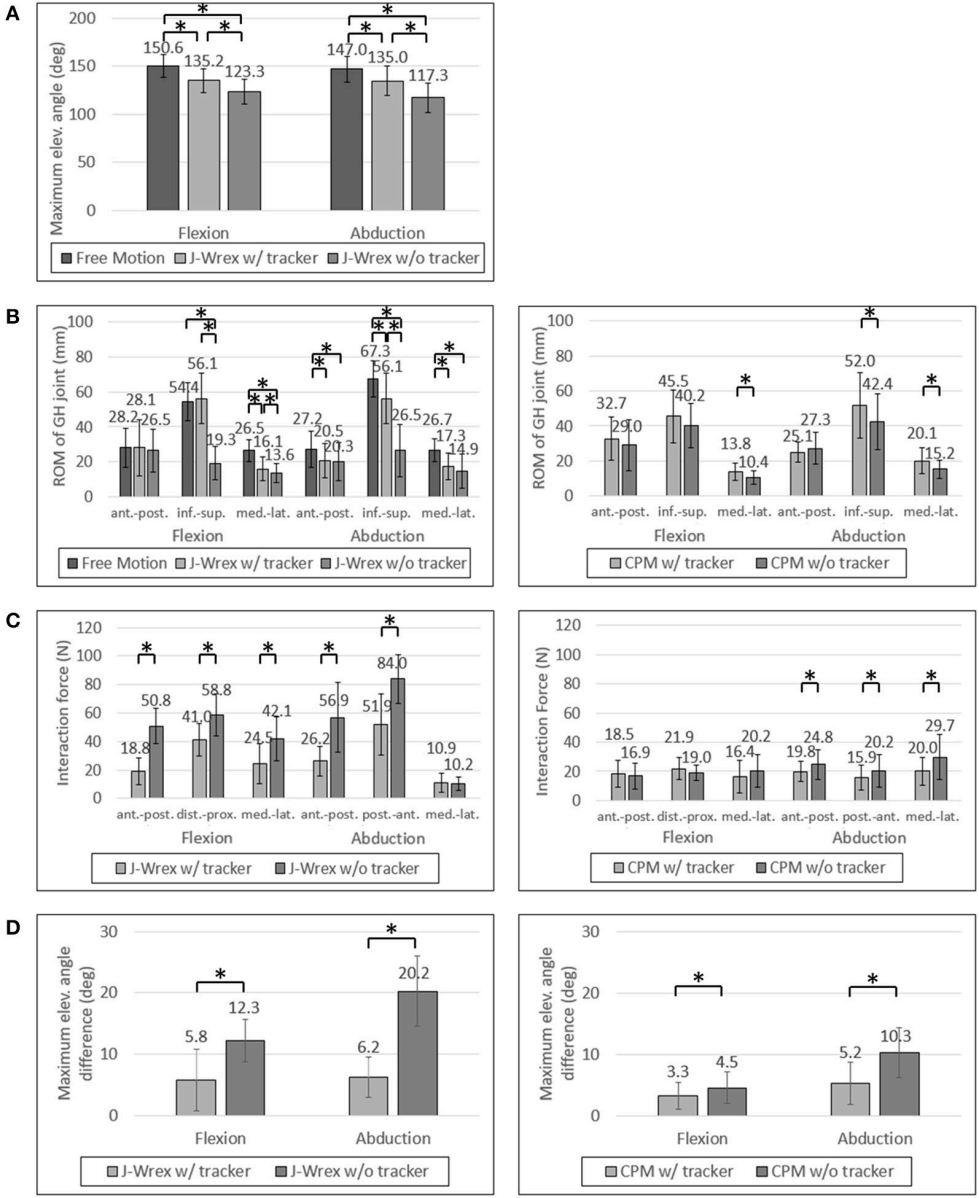
Experimental data of healthy subjects: **(A)** Maximum elevation angle, **(B)** ROM of GH joint, **(C)** Interaction Force and **(D)** Angle difference between device and arm. Asterisk means statistical difference (*p*-value < 0.05).

The experimental results showed that the ROM of the w/ tracker condition was larger than that of the w/o tracker condition and the difference was statistically significant (Figure 7A). It was more natural for the healthy subjects that they did not wear a rehabilitation device when raising their arms. As the maximum angle of the J-Wrex was limited to ~150°, the maximum angle of the arm is lowered even if the tracker allowed translational movement of the shoulder joint.

All the healthy subjects were able to raise their arms by more than 150° but we set the maximum angle to 150°; therefore, the maximum angle was not compared under the CPM condition.

**Figure 8:**
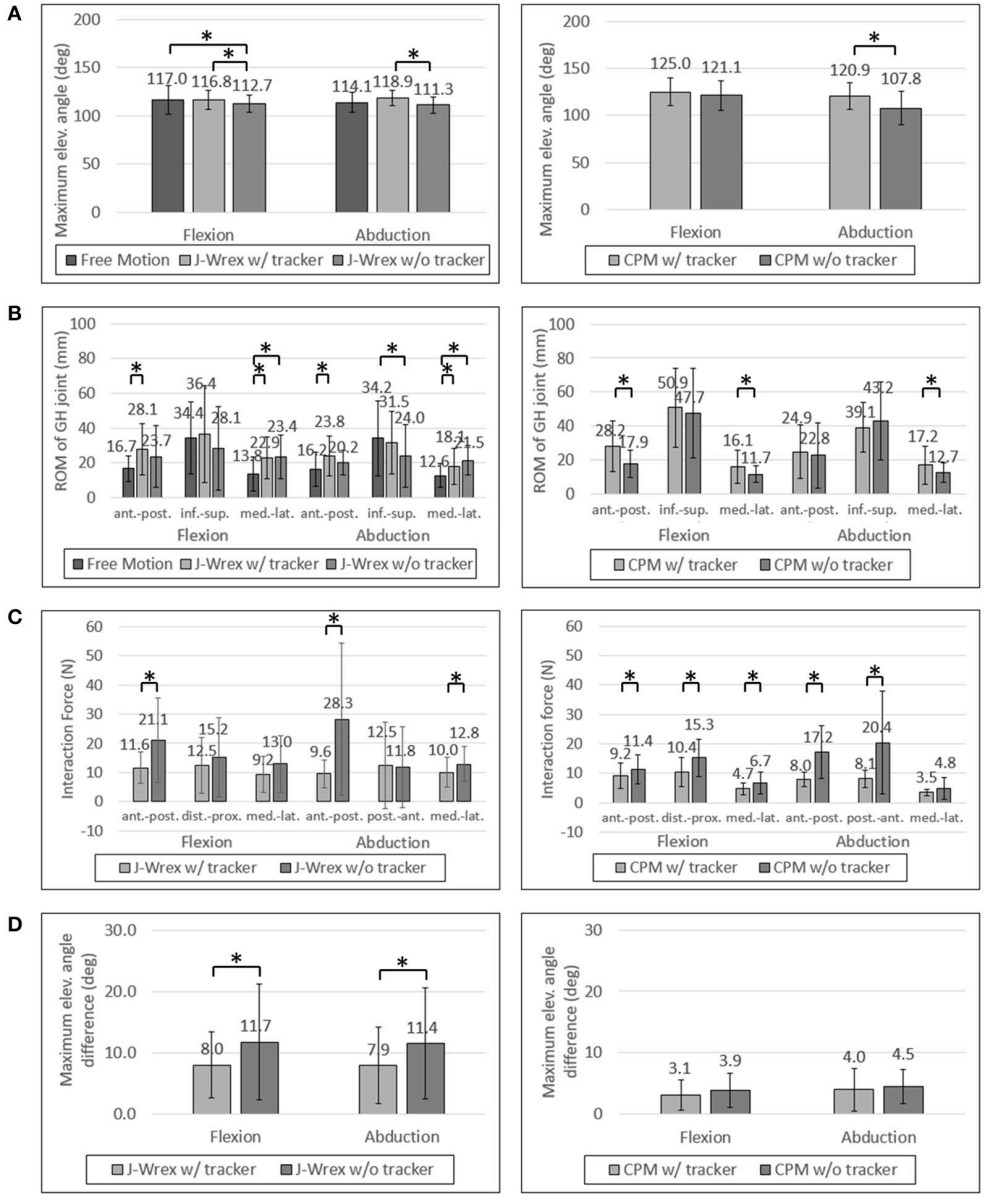
Experimental data of patients: **(A)** Maximum elevation angle, **(B)** ROM of GH joint, **(C)** Interaction Force and **(D)** Angle difference between device and arm. Asterisk means statistical difference (*p*-value < 0.05).

The maximum angle data in the J-Wrex experiment were analyzed for six patients, excluding the two patients whose ROM was almost similar to that of the healthy subjects. Under the w/ tracker condition, the patients could raise their arms by almost as much as it would be possible during free motion. The maximum elevation angle was lower than the angle of free motion under the w/o tracker condition (Figure 8A). There is a significant difference between the w/ tracker and w/o tracker conditions in flexion motion. In abduction motion, the J-Wrex combined with the tracker effectively increased the maximum elevation angle (*p* < 0.05).

In the CPM experiment, patients were able to raise their arms higher under w/ tracker conditions than under w/o tracker conditions (Figure 7A). The elevation angle was significantly increased in abduction motion (*p* < 0.05).

### ROM of the GH joint

The ROM of the GH joint was analyzed only to determine the ROM of the arm elevation shared among the three conditions: free motion, w/ tracker, and w/o tracker.

In healthy subjects and patients, the ROM in the inferior–superior direction was larger than the ROM in the transversal direction, that is, the medial–lateral and anterior–posterior directions (Figures 7B, 8B). The ROM of free condition and the w/ tracker condition were almost same, whereas that of the w/o tracker condition was mostly small.

There was no significant difference in the transversal ROM of the GH joint in the J-Wrex experiment, except for the free motion condition. The inferior–superior ROM was the largest for the w/ tracker condition and the free motion condition in flexion and abduction, respectively. It was the smallest when using only J-Wrex for both the motions.

In the CPM experiment, the ROM of the shoulder joint increased for the w/ tracker condition at flexion motion, and there was a significant difference for the w/o tracker condition in the transversal direction (*p* < 0.05). In the abduction motion, only the medial–lateral ROM increased significantly when using the tracker, but there was no significant difference in the other directions.

Although the ROM of the rehabilitation operation was also important, when the patients performed the operation, they experienced an unintended force causing them to become uncomfortable. Therefore, this force should be analyzed as well.

### Interaction force

The effect of comfort on the tracker was determined by analyzing the force applied to the shoulder joint. The analysis entailed comparing the force between the w/ tracker and w/o tracker conditions.

In healthy subjects, the J-Wrex experiment showed significant decrease in force, except for the medial–lateral direction in the abduction (Figure 7C). The anterior–posterior force decreased to 37% and 46% in flexion and abduction, respectively. In the CPM experiment, there were no significant differences between the two device conditions in the flexion, whereas there were significant differences in the abduction. There was little difference in force because there was no difference between the shoulder movements.

In the patient group, the maximum force exerted on the shoulder joint in the J-Wrex experiment was significantly different in the anterior–posterior direction of the two motions (Figure 8C). In the flexion motion, the force decreased to 55%, 82%, and 71% in the anterior–posterior, distal–proximal, and medial–lateral directions, respectively. In the abduction motion, there was almost no difference in the distal–proximal direction, and the force decreased to 34% and 78% in the anterior–posterior and medial–lateral direction, respectively. In both the motions, the force in the anterior–posterior direction was greatly reduced, indicating that the force was particularly reduced in the direction perpendicular to the sensor.

In the CPM experiments with the patient group, the force measured in both the motions showed a significant difference in both the anterior–posterior and the distal–proximal directions. In flexion motion, the maximum force was reduced to 81%, 68%, and 70% in the anterior–posterior, distal–proximal, and medial–lateral directions, respectively. In the abduction motion, it decreased significantly to 46% and 40% in the anterior–posterior and distal–proximal directions, respectively.

As the interaction force is the most important factor among the parameters, we also analyzed the interaction force of two patients group: central nervous system (CNS) group and shoulder pain due to fracture group. Patients using the CPM device were classified as having diseases associated with the CNS (patients 1, 4, and 5) and shoulder pain due to fracture (patients 2, 3, 6, 7, 8, and 9). In the CNS patients, the force was reduced to almost half of the distal–proximal force and decreased to less than 44% in all directions for abduction. In the shoulder stiffness group, the force decreased to 63% in the medial–lateral direction of flexion motion and it was 54% and 49% in the distal–proximal and anterior–posterior directions, respectively, in abduction motion, when attached to the tracker. This result indicated that CNS patients who had difficulty in raising their arms on their own could benefit from rehabilitation by using the tracker.

In the J-Wrex experiment, only one patient had CNS disease (patient 5), and patients without shoulder stiffness also participated. Therefore, patients were divided into patients with shoulder stiffness (patients 2, 3, 6, 8, and 11) and those without stiffness (patients 5, 9, and 10). Patients with shoulder stiffness showed a significant difference in anterior–posterior direction and the force decreased to 61.2% and 56.7% for flexion and abduction, respectively. In the group without shoulder stiffness, there was no statistically significant difference in all directions, but the force in the anterior–posterior decreased to 49.5% and 26.0% in flexion and abduction, respectively. Regardless of the shoulder stiffness, the force in the normal direction of the sensor was reduced because J-Wrex did not allow movement of the shoulder joint in the vertical direction.

### Angle difference between device and arm

For healthy subjects, the angle difference, which caused a substantial difference in strength, was significant in the experiments in which both the J-Wrex and the CPM (Figure 7D) were used.

For the patient group, the maximum angular difference was reduced to ~70% with a significant difference when using the tracker in both flexion and abduction (Figure 8D). In the CPM experiment, the maximum angular difference was reduced to 81% and 90% when using the tracker in flexion and abduction, respectively, but the result is not statistically significant.

## Discussion and conclusion

In this study, we developed a passive shoulder tracking module that allowed tracking of the natural GH movement during upper extremity rehabilitation. As the module was designed to be augmented to the existing upper-limb devices, we verified the performance of the device with the two commercially available rehabilitation devices, such as J-Wrex and CPM. The GH joint tracking performance was experimentally validated. As a result, the subjects could raise their arms higher while feeling less resisting force from the device when the commercial devices were combined with the proposed tracking module. The addition of the tracking module would enable more comfortable and effective shoulder rehabilitation, by achieving greater ROM with less interaction force for the same rehabilitation task.

In the CPM experiments with healthy subjects, experiments were performed in the range of 45°–150°. Because of the limited maximum angle, there was no significant improvement.

The ROMs of the GH joint in the transverse plane (anterior–posterior and medial–lateral directions) were not significantly improved because J-Wrex allowed tracking of the GH joint in part by the additional horizontal revolute joint in commercial J-Wrex.

Even though the vertical positions of the devices were fixed in the “w/o tracker” condition, there is a clearance between the brace and the patient's arms, which allows vertical movement of the GH joint. This clearance is needed for comfortable fitting in the subjects as very tight fitting caused pain. Therefore, the clearance might have caused no significant difference in the vertical GH joint movement because the arm could be slid or tilted inside the brace; however, it should be noted that the increased clearance accompanies additional force between the brace and the subject's arm, which can be seen in the significant increase of the interaction force in the “w/o tracker” condition. The clearance can also be seen in the difference between the device joint angle and the arm joint angle. The angular difference between the device and the arm was relatively large in the J-Wrex experiments because the subjects conducted active movement with larger clearance. Therefore, the angular difference and the interaction force are relatively larger in J-Wrex, which implies that the subjects strongly overcame the resistance from the device. In the CPM, however, the angular differences were relatively smaller because the subjects relaxed and leaned their arms on the brace during passive stretching.

Overall, using the device in combination with a tracker reduced the interactive force and the clearance between the device and the upper arm. This is expected to reduce the burden on the subjects and allow more accurate exercise when it is performed with the same rehabilitation device.

As only patients who were able to raise their arms by more than 120° were recruited, only a few patients experienced this condition. Therefore, recruiting more patients would yield more reliable results that are statistically significant.

In this experiment, a light tracking device was used when combining with the J-Wrex to increase the follow-up performance. The passive tracking device used a constant-force spring to lift the rehabilitation device and the patient's arm weight. This approach requires the load of the counter mass or the load of the constant-force spring to be changed for each subject. It can be inconvenient in clinics where many patients need to be treated in a short time. In addition, when manufacturing a device with a high-rated load with consideration for all devices and patients, the inertia of the device may lower the tracking performance. Therefore, if a mechanism to adjust the compensating load for each subject and device is added, then the passive shoulder joint tracking module can be used more extensively in clinics.

## Author contributions

K-SL, J-HP, and H-SP designed the device. K-SL, J-HP, JB, and H-SP conceived and planned the experiments. K-SL, and J-HP carried out the experiment and analyzed the data, and H-SP supervised whole procedures. K-SL and J-HP wrote the manuscript in consultation with JB and H-SP.

### Conflict of interest statement

The authors declare that the research was conducted in the absence of any commercial or financial relationships that could be construed as a potential conflict of interest.
